# Density-Adjustable Bio-Based Polysulfide Composite Prepared by Inverse Vulcanization and Bio-Based Fillers

**DOI:** 10.3390/polym12092127

**Published:** 2020-09-18

**Authors:** Yanxia Liu, Yidan Chen, Yagang Zhang, Yurong Chen, Lulu Wang, Xingjie Zan, Letao Zhang

**Affiliations:** 1 Xinjiang Technical Institute of Physics and Chemistry, Chinese Academy of Sciences, Urumqi 830011, China; liuyanxia@ms.xjb.ac.cn (Y.L.); chenyd2020@ms.xjb.ac.cn (Y.C.); chenyurong@ms.xjb.ac.cn (Y.C.); wanglulu@ms.xjb.ac.cn (L.W.); zhanglt@ms.xjb.ac.cn (L.Z.); 2 University of Chinese Academy of Sciences, Beijing 100049, China; 3 Department of Chemical and Environmental Engineering, Xinjiang Institute of Engineering, Urumqi 830026, China; 4 School of Materials and Energy, University of Electronic Science and Technology of China, Chengdu 611731, China

**Keywords:** sulfur, cottonseed oil, density-adjustable, inverse vulcanization, polysulfide composites

## Abstract

Excess sulfur has become a global problem in petrochemical industry. Inexpensive and easily available cottonseed oil (CSO) is still underutilized. To resolve these issues, bio-based polysulfide composites were prepared via inverse vulcanization of sulfur and CSO. The density of polysulfide composites was adjusted by fillers. The results showed that Elm and cattail as the fillers had no effects on the thermal properties and chemical structures of polysulfide composites. However, the morphologies of polysulfide composites were significantly influenced by the fillers. Different types and amounts of fillers produced significantly different holes and folds in the composites. The fillers were embedded in polysulfide composites by physical filling. This study provides an alternative and promising approach for preparing affordable density-adjustable bio-based polysulfide composite.

## 1. Introduction

Sulfur (S) as a byproduct of petroleum refining, gas reserves, and other petrochemical industry, being produced in excess of 70 million tons each year. Sulfur is widely used in commodity chemicals, such as sulfuric acid, fertilizers, and rubbers [1,2,3]. However, these traditional applications cannot consume excessive amount of sulfur. There are still about 10 million tons of sulfur surplus every year, creating large unwanted stockpiles and a global issue “excess sulfur problem” in petrochemical industry [4,5]. Therefore, it is a promising way to effectively utilize sulfur to develop value-added polymer materials [6,7,8,9].

Orthorhombic sulfur (S_α_) is the most thermodynamically stable form of elemental sulfur. S_α_ presents in the form of eight-membered rings (S_8_) [10]. The heating of S_α_ leads it to be converted into monoclinic sulfur (S_β_, S_8_) at 95.6 °C. Further heating, solid sulfur (mainly S_β_ and residual S_α_) melts, and yellow liquid sulfur (S_λ_, S_8_) was obtained at melting point temperature of 119 °C. When the temperature continues to rise to 159 °C, sulfur S_8_ rings opening polymerization forms the deep red liquid sulfur (S_μ_). S_μ_ continues to spontaneously polymerize, resulting in the formation of linear sulfur radical high polymer (S_w_) [6,10,11,12,13,14].

Free radical polymerization of sulfur with compound containing unsaturated bonds has been investigated as a good means of generating sulfur-based polysulfide. The reaction between sulfur and unsaturated compound is known as “inverse vulcanization” [5]. Polysulfides usually exhibit excellent properties preferring them suitable for direct use or as intermediates for further functionalization for various applications [15,16] such as solar cell [17], hydrogen fuel cell [18], lithium-ion batteries [11,19,20,21,22,23,24,25,26], high refractive index and IR transmitting optical devices [27,28,29,30], sulfur-doped carbon materials [31], heavy metal remediation [14,31,32,33,34,35], rubber [10], fertilizer [36,37], and other advanced materials [38,39,40].

The majority of polysulfides are obtained via the copolymerization of sulfur with vinylic monomers. Vinylic monomers are mainly derived from non-renewable petroleum resources. Therefore, the development of bio-based renewable monomers is very important in practice [41]. Bio-based polysulfides are desirable new chemical materials that not only help solve environmental pollution problems, but also achieve a sustainable future. It would be ideal to replace petroleum-based monomers with cheaper, renewable bio-based resources to reduce the increasing consumption of fossil fuels [42].

There is a growing demand to develop inexpensive bio-based polymers to replace conventional petroleum-based polymers [41]. Recently, researchers began to use vegetable oils as vinylic monomers to develop bio-based polysulfides [32,36,43,44,45]. The main component of vegetable oil is triglyceride. The alkene functional groups in triglyceride aliphatic chain provide the necessary crosslinking points for inverse vulcanization. It has been reported that the *Z* stereochemistry of these alkenes, imparting strain to the olefin, would conducive to rapid react with the sulfur free radicals generated in the process of inverse vulcanization [32]. The reaction mechanism of vegetable oil with sulfur to prepare polysulfide is as follows. The C=C double bonds present in the triglyceride of vegetable oil break to react with ring-opened sulfur leading the formation of cross-linked polysulfide at a certain temperature [41].

Theato et al. prepared bio-based polysulfides via vegetable oils (linseed oil, sunflower oil and olive oil) and sulfur [43]. The properties of these polymers as cathode materials for “green Li-S batteries” were studied. SEM showed that the composite materials consist of micron-sized sulfur particles embedded in a cross-linked polymeric network. The polymeric network formed by copolymerization of fatty chain residues with sulfur was similar to factice. This was the first time to use polysulfide as the active cathode material of Li-S batteries. Chalker et al. have done a series of work on bio-based polysulfides [32,44,46]. Chalker et al. used unsaturated cooking oils to prepare polysulfide. The polysulfide was used as low-cost mercury sorbents [32]. In addition, Chalker et al. set out to assess the iron-binding properties of polysulfide prepared via the reaction of canola oil and sulfur. They found that the porous polysulfide was superior in the rate of Fe^3+^ removal from water [44]. Furthermore, Chalker et al. prepared polysulfide adsorbent to remove hydrocarbons via cooking oil and sulfur. The polysulfide had an affinity for hydrocarbons such as crude oil and diesel fuel that can be quickly removed from seawater, and the polysulfide showed good recovery and multiple reusability [46]. Ribeiro et al. used soybean oil as monomer to prepare a new fertilizer. The results showed that S_8_ structure is more accessible to oxidizing microorganisms by chemical modification via inverse vulcanization [36]. Wang et al. synthesized a polysulfide using sulfur and transgenic soybean oil. Gel permeation chromatography results indicated that the polysulfide was a hyperbranched polymer and it is potential green plasticizers for plastics or elastomers [45].

In summary, the preparation of environmentally friendly bio-based polysulfides using vegetable oils and sulfur has attracted considerable attention. The polysulfides have broad application prospects. Cotton is the ninth largest oil-producing crop [47,48,49]. Cottonseed oil (CSO) is extracted from the seeds of cotton plant after removal of cotton lint. CSO contains significant amounts of saturated fatty acids palmitic acid (22–26%), lesser amounts of monounsaturated fatty acids oleic acid (15–20%), diunsaturated linoleic acid (49–58%) being the most prominent fatty acid [49]. CSO has high production and lower price than other oils, such as soybean oil, canola oil, and peanut oil. However, cheap and easily available CSO still cannot take advantage of high value [50]. 

In this study, the raw material sulfur is a by-product of petrochemical industry and CSO is cheap and has excellent biodegradability. The preparation of polysulfide using sulfur and vegetable oil is an innovative example of green chemistry and waste valorization. Furthermore, this approach helps increasing atom economy and requires no solvent. In order to broaden the application of polysulfide, tuning the density of polysulfide is extremely important for practical applications. Along these lines, the density-adjustable polysulfide composites were designed with bio-based fillers, elm and cattail. The results showed that the fillers were crucial for regulating the density of polysulfide composites. To the best of our knowledge, our work is the first attempt to adjust and control the density of polysulfide composites, and systematically studying the effects of fillers on the density and structure of polysulfide composites. This study has important theoretical and practical significance for the resource utilization and the development of value-added products with sulfur and vegetable oil.

## 2. Materials and Methods

### 2.1. Materials

Sulfur and active carbon were purchased from Tianjing Baishi Chemical Industry Co. Ltd., (Tianjing, China). Cottonseed oil (CSO) was purchased from Xinjiang Sayram Modern Agriculture Co. Ltd., (Bole, Xinjiang, China). Active clay was purchased from Jiangsu Goldenstone Attapulgite Minng Industry Co. Ltd., (Huaian, Jiangsu, China). We purchased 300–800 carboxymethyl cellulose (CMC) from Shanghai Shanpu Chemical Co. Ltd., (Shanghai, China). Elm from the samara of the elm tree was collected from Xinjiang Technical Institute of Physics and Chemistry, China (Urumqi, Xinjiang, China). Cattail from the fruit part of the plant was collected from Bortala River, China (Bole, Xinjiang, China). Elm and Cattail were cleaned with distilled water and dried in a vacuum drying oven at 60 °C until constant weight. The dried materials were pulverized and screened to prepare bio-based polysulfide composites.

Characteristics of the fillers are as follows. Active carbon is a kind of black porous solid carbon with a hydrophobic surface and a specific surface area of 800–1200 m^2^ g^−1^. Active clay is an adsorbent with high activity which is processed from bentonite. The chemical formula of active clay is Al_2_O_3_·4SiO_2_·nH_2_O, which contains a large number of exchangeable cations. Carboxymethyl cellulose (CMC) is an anionic cellulose ether, and its degree of polymerization affects the viscosity of the product. In this study, CMC with viscosity of 300–800 mPa s and 800–1200 mPa s were selected and tested. Elm and cattail are mainly composed of cellulose, hemicellulose, and lignin. Cellulose is a macromolecular polysaccharide composed of glucose. Hemicellulose is a polyphase polymer composed of several different types of pentose and hexose. Lignin is an aromatic high polymer whose molecular structure contains phenylpropanol and its derivatives.

### 2.2. Synthesis of Bio-Based Polysulfide Composite

**Scheme 1:**
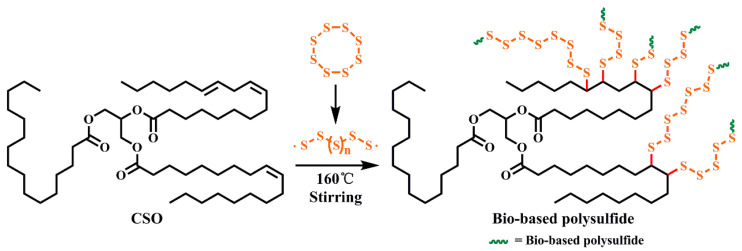
Synthesis route of bio-based polysulfide.

Firstly, a certain amount of sulfur was added to a 100 mL glass beaker equipped with a magnetic stir, and heated to 160 °C in a thermostated oil bath until a molten phase was formed. Then 10.0 g CSO was added dropwise, resulting in a two-phase mixture. The mixture was stirred vigorously to ensure efficient mixing. After one phase was formed, a certain amount of filler was added gradually. Heating was continued at 160 °C to make the reactant form a uniform liquid. Along with the extension of time, the viscosity of the polymer gradually increased. When the polymer became solid, the reaction stopped. The beaker was taken out to cool down naturally, and the bio-based polysulfide composite was obtained. Active carbon, active clay, CMC, elm and cattail were chosen as filler materials. Synthesis route of bio-based polysulfide is shown in Scheme 1. 

### 2.3. Density and Hardness Determination of Bio-Based Polysulfide Composite

XF-120MD (Xiamen, Fujian, China) digital densitometer was used to determine the density of bio-based polysulfide composite. LX-A Shore durometer (Shanghai, China) was used to measure the hardness of bio-based polysulfide composite.

### 2.4. Characterizations

Morphological analysis was carried out on a SUPRA 55vp ZEISS field emission scanning electron microscopy (Oberkochen, Germany), while element mapping were obtained using a corresponding energy dispersive X-ray (EDX) spectra. Elemental analysis was carried out on Elementar vario MICRO cube elemental analyzer (Frankfurt, Germany). Carbon, hydrogen, nitrogen, and sulfur content (% CHNS) was determined by combustible analysis using a thermal conductivity detector. Differential scanning calorimetry (DSC), thermogravimetry analysis (TGA), and derivative thermogravimetry (DTG) were heated under nitrogen to 800 °C at a heating rate of 10 °C min^−1^ using a simultaneous thermal analyzer Setaram Labsys Evolution (Lyons, France). Fourier-transform infrared spectroscopy (FT-IR) was performed using a Bruker VERTEX-70 FT-IR spectroscopy (Ettlingen, Germany), between 650 cm^−1^ to 4000 cm^−1^. Raman spectra was acquired using a Horiba Scientific Raman microscope (Paris, France) at an excitation laser wavelength of 532 nm with a 50X objective.

## 3. Results and Discussion

### 3.1. Synthesis of Bio-Based Polysulfide Composite

**Figure 1:**
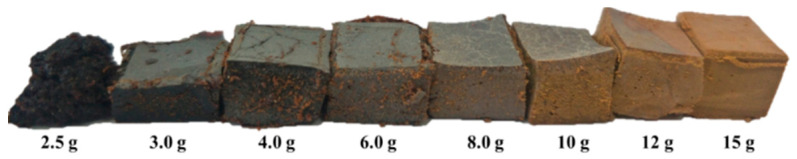
Picture of bio-based polysulfides prepared with different sulfur content.

Chemically stable bio-based polysulfides were obtained via inverse vulcanization of sulfur (S_8_) with CSO, the rubbery-like polysulfides are shown in Figure 1. No solvents or exogenous reagents were needed in the synthesis process [32]. Table 1 depicts that the amount of sulfur was a key factor affecting the formation of bio-based polysulfides, determining the density, hardness and gelation point of polysulfides. When the mass ratio of CSO to sulfur was too small (10.0 g:2.0 g), the solid polysulfide cannot be formed. With the increase of sulfur content, the density and hardness of the polysulfides increased and the gelation time shortened. However, due to the high sulfur content (over 10.0 g), the polysulfides had holes on the surface and inside, resulting in uneven and fragile polysulfides. This was attributed to the variation in excessive sulfur content. Some residual sulfur did not participate in inverse vulcanization. These results were confirmed by Wręczycki [10] and Valle et al. [36]. The results showed that the amount of sulfur had a great influence on the appearance of bio-based polysulfides. The polysulfides with low sulfur content were dark in color. As the amount of sulfur increased, the color of the polysulfides gradually changed from black to light brown.

10.0 g CSO + 10.0 g sulfur system was selected to study the effects of different types of fillers on the density of bio-based polysulfide composites. This was considered appropriate ratio according to the density, hardness, and gelation time of the polysulfides composites. Moreover, sulfur content was relatively higher, which was desirable for the resource utilization. Active carbon, active clay, CMC, elm and cattail as fillers were examined and screened (Table 2). Table 2 shows that active carbon, active clay and CMC had little effect on the density of polysulfide composites. Elm and cattail made the density of polysulfide composites decreased. Elm and cattail are cheap and readily available, and they had a relatively great influence on the density of polysulfide composites, so they were chosen as the density-controlling materials of polysulfide composites in this study.

**Figure 2:**
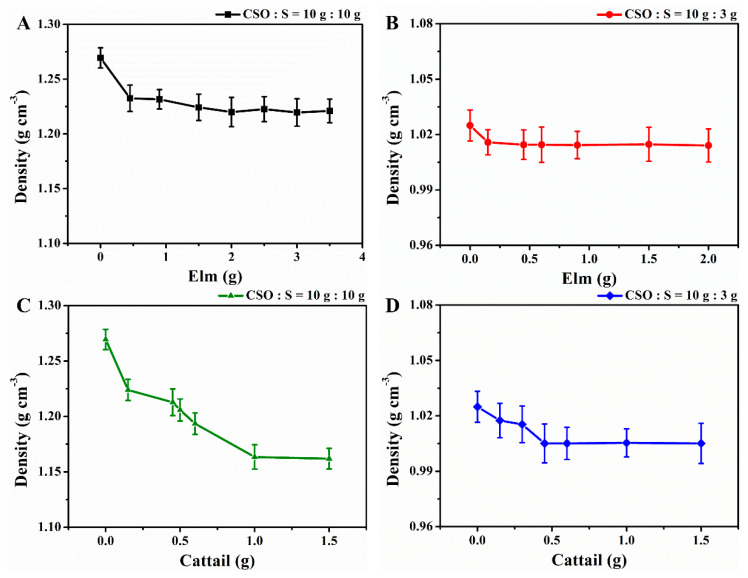
Effect of filler content on density of bio-based polysulfide composites. (**A**) 10.0 g CSO + 10.0 g S + elm, (**B**) 10.0 g CSO + 3.0 g S + elm, (**C**) 10.0 g CSO + 10.0 g S + cattail, (**D**) 10.0 g CSO + 3.0 g S + cattail.

**Figure 3:**
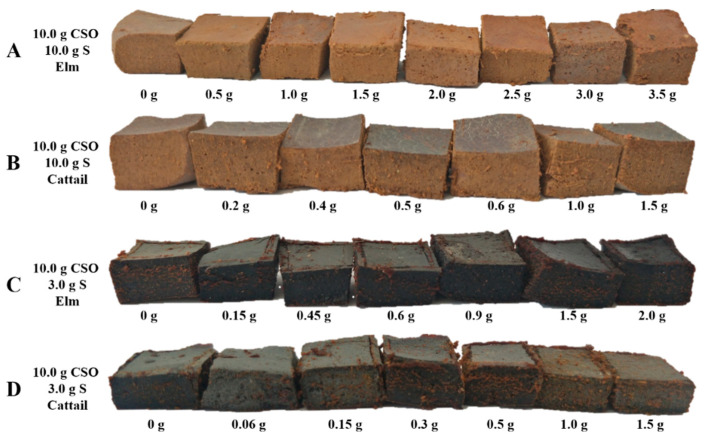
Picture of bio-based polysulfide composites prepared with different fillers. (**A**) 10.0 g CSO + 10.0 g S + elm, (**B**) 10.0 g CSO + 10.0 g S + cattail, (**C**) 10.0 g CSO + 3.0 g S + elm, (**D**) 10.0 g CSO + 3.0 g S + cattail.

One important application of polysulfide composites is in water. Density is a key performance to determine the effectiveness of its use. The density of polysulfide composites should be close to that of applied water. Therefore, this study also explored 10.0 g CSO + 3.0 g sulfur system, the density of polysulfide was 1.02 g cm^−3^ (Table 1). Consequently, 10.0 g CSO + 10.0 g sulfur system and 10.0 g CSO + 3.0 g sulfur system were selected to study the regulatory ability of bio-based fillers (elm and cattail) on the density of polysulfide composites (Figure 2 and Figure 3).

Figure 2 shows that the effects of fillers content on the densities of polysulfide composites were consistent in different CSO and sulfur system. With the increased of fillers content, the density of polysulfide composites decreased first and then remained unchanged. The densities of polysulfides prepared by 10.0 g CSO + 10.0 g sulfur and 10.0 g CSO + 3.0 g sulfur were 1.27 g cm^−3^ and 1.02 g cm^−3^, respectively (Table 1). While the densities of elm and cattail were 0.48 g cm^−3^ and 0.32 g cm^−3^, respectively. 

The results showed that the density of polysulfide composites decreased first and then remained unchanged with the increased amount of fillers. Noticeably, elm and cattail are both materials with loose structure and low density, the density of the prepared polysulfide composite will decrease when the amount of filler increased only if the simple physical mixture occurred. However, the preparation of polysulfide composites involves chemical crosslinking reactions as well as expel and trap of air in cavities and channels within both plant rubber and filler materials. As the results of both reasons, so the density of polysulfide composites decreased with the increase of filler within a certain range. However, when the amount of filler further increased, the density of polysulfide composites was basically unchanged. This is due to more polysulfide penetrate the pores and replace the air pores during the preparation of the composite. When the pores and cavities inside the composite are filled with polysulfide, the density of the polymer composite does not change too much. The results showed that addition of appropriate amount of elm and cattail could adjust the density of polysulfide composites within a certain range.

Figure 3 shows that the contents of sulfur had a great influence on the appearance of bio-based polysulfide composites. When the sulfur content was higher, the polysulfide composite was light in color. While, when the sulfur content was lower, the color of polysulfur composite was dark. Figure 3A,B shows that the colors of polysulfide composites prepared by 10.0 g CSO + 10.0 g sulfur system were nearly brown. This was due to sulfur was excessive, there were residual sulfur particles inside and on the surface of bio-based polysulfide composites. Moreover, when the amount of elm was more than 3.0 g and cattail was more than 1.5 g, fillers were attached with the polysulfide composites. The same situation also occurred in 10.0 g CSO + 3.0 g sulfur system, the colors of polysulfide composites were nearly black (Figure 3C,D). When the amount of elm was more than 1.5 g and cattail was more than 0.5 g, fillers were also attached with the polysulfide composites. Therefore, the content of fillers should be controlled within a certain range when regulated the density of bio-based polysulfide composites.

### 3.2. Characterization

#### 3.2.1. SEM and EDX

**Figure 4:**
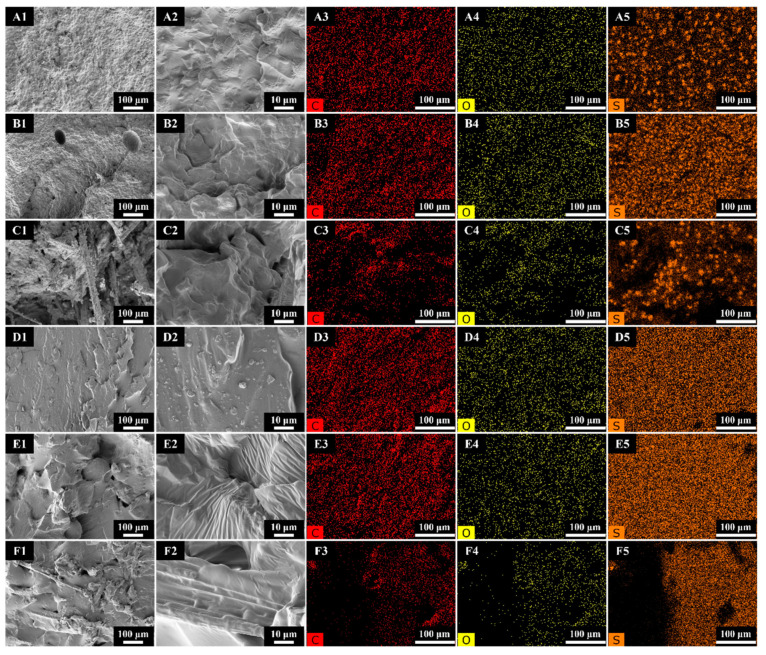
SEM image and element mapping of bio-based polysulfide composite. (**A1**–**A5**) 10.0 g CSO + 10.0 g S, (**B1**–**B5**) 10.0 g CSO + 10.0 g S + 2.0 g elm, (**C1**–**C5**) 10.0 g CSO + 10.0 g S + 1.0 g cattail, (**D1**–**D5**) 10.0 g CSO + 3.0 g S, (**E1**–**E5**) 10.0 g CSO + 3.0 g S + 0.45 g elm, (**F1**–**F5**) 10.0 g CSO + 3.0 g S + 0.3 g cattail.

The bio-based polysulfide composites were analyzed by SEM and EDX to assess the surface morphology and elemental composition. The results are shown in Figure 4. The polysulfide composites presented different morphology characteristics with different sulfur content and fillers.

Figure 4A1,A2 shows that the surface of polysulfide prepared by 10.0 g CSO and 10.0 g sulfur was uniform and rough, with the appearance of fish scales. Figure 4B1,B2 shows that the surface of elm-added polysulfide composite was also uniform and rough, like fish scales, but there were a few holes and cracks in the polysulfide composite. But, the surface of cattail-added polysulfide composite was completely different (Figure 4C1,C2). The surface morphology was uneven, the structure was complex, and there were many holes in cattail-added polysulfide composite. The filler cattail can be seen clearly in Figure 4C1,C2. These results confirmed that elm and cattail can reduce the density of polysulfide composite. Compared with the polysulfide prepared by 10.0 g CSO and 10.0 g sulfur, the polysulfide prepared by 10.0 g CSO and 3.0 g sulfur had a uniform and smooth surface with ridges and folds, similar to mountains (Figure 4D1,D2)). This proved that the density of later polysulfide composite was relatively lower than that of former. Figure 4E1,E2 shows that the surface of elm-added polysulfide composite was uneven and irregular with abundant holes and folds. While the surface of cattail-added polysulfide composite was more uneven and irregular with abundant holes and folds and the filler cattail also can be clearly seen in cattail-added polysulfide composite (Figure 4F1,F2). These results suggested that elm and cattail can reduce the density of polysulfide composite. We speculated that elm and cattail might be embedded in the polysulfide composites by physical filling to achieve the goal of regulating the densities of polysulfide composites.

The elemental mappings show the spatial distribution of carbon, oxygen and sulfur. Figure 4A3–A5,B3–B5,C3–C5 shows that carbon and oxygen elements were uniformly dispersed in bio-based polysulfide composites in 10.0 g CSO + 10.0 g sulfur system. The aggregation of sulfur element shows that raw material sulfur was excessive. The presence of unreacted sulfur in polysulfide composites made the material brittle. Figure 4D3–D5,E3–E5,F3–F5 shows that carbon, oxygen and sulfur elements were homogeneously distributed in the polysulfide composites in 10.0 g CSO + 3.0 g sulfur system. This indicated that the polysulfide composites were homogeneous in structure and sulfur was fully involved in inverse vulcanization reaction.

#### 3.2.2. Elemental Analysis

The element contents of bio-based polysulfide composites were analyzed by an element analyzer (Table 3). The contents of S, C, H and N in polysulfide prepared in 10.0 g CSO + 10.0 g sulfur system were 52.70%, 38.02%, 5.57%, and 0.07%, respectively, consistent with the high sulfur content envisioned for this product. The results were basically consistent with Crockett et al., elemental composition of polymer were 56.6% S, 38.97% C, and 4.97% H [14]. The contents of S, C, H and N in bio-based polysulfide prepared in 10.0 g CSO + 3.0 g sulfur system were 23.31%, 59.49%, 8.48%, and 0.05%, respectively. Theoretically, adding fillers to the system can reduce the content of S element in polysulfide composites. Table 3 shows that both in 10.0 g CSO + 10.0 g sulfur and 10.0 g CSO + 3.0 g sulfur system, the contents of S element in polysulfide composites decreased slightly after adding elm and cattail. The results were consistent with the theory.

#### 3.2.3. DSC

**Figure 5:**
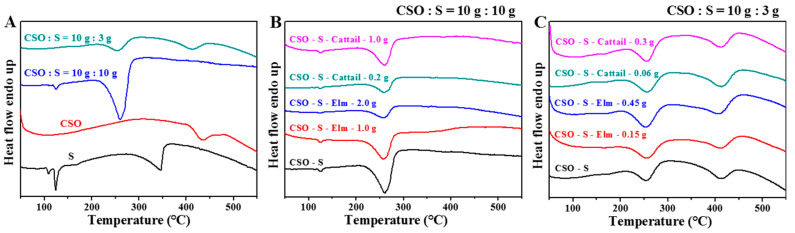
DSC thermograms of sulfur, cottonseed oil (CSO) and bio-based polysulfide composites. (**A**) S, CSO, 10.0 g CSO + 10.0 g S, 10.0 g CSO + 3.0 g S, (**B**) 10.0 g CSO + 10.0 g S + different fillers, (**C**) 10.0 g CSO + 3.0 g S + different fillers.

Differential scanning calorimetry (DSC) analysis was conducted to investigate the phase structures of bio-based polysulfide composites. The comparison of DSC thermograms is presented in Figure 5. Sulfur exhibited three endothermic peaks during heating (Figure 5A). The first peak occurred at approximately 109 °C and corresponded to the change in crystalline structure of sulfur from orthorhombic (S_α_) to monoclinic (S_β_). The second one, which occurred at approximately 120 °C, was related to the melting of S_β_ crystals and residual S_α_ crystals into yellow liquid (S_λ_). The third peak occurred during approximately 200–360 °C. This indicated S might undergo a phase change along with evaporation and sublimation at that temperature. Liquid sulfur was converted to gaseous sulfur. During heating, CSO exhibited one endothermic peak during near 280–510 °C, the phase structure of CSO began to change, CSO could also decompose to produce other substances. Similar DSC thermograms were observed in bio-based polysulfide composites with and without fillers (Figure 5B,C). This was due to the contents of fillers in vulcanized composites were too low, which had no effects on the phase transition of the polysulfide composites. Bio-based polysulfide composites prepared in 10.0 g CSO + 10.0 g sulfur system showed two endothermic peaks, approximately 125 °C and during 200–300 °C, respectively (Figure 5B). The first peak indicated that there were incomplete sulfur in polysulfide composites, which was caused by the phase transition of sulfur. The second peak was the phase transition peak of bio-based polysulfide composites. The results indicated that the unreacted sulfur remained in the polysulfide composites. The polysulfide composites prepared in 10.0 g CSO + 3.0 g sulfur system also showed two endothermic peaks, during approximately 200–300 °C and 300–500 °C, respectively (Figure 5C). The first peak was the phase transition peak of polysulfide composites. The second peak was the phase transition peak of residual COS. The results indicated that the unreacted CSO remained in the polysulfide composites. DSC thermograms proved that fillers did not affect the phase transition of bio-based polysulfide composites. 

**Figure 6:**
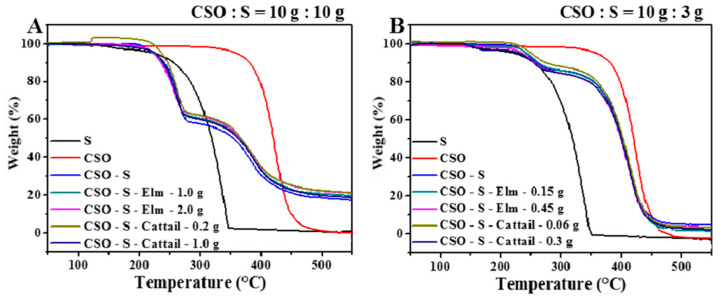
Thermogravimetric analysis (TGA) curves of sulfur, cottonseed oil (CSO) and bio-based polysulfide composites. (**A**) 10.0 g CSO + 10.0 g S + different fillers, (**B**) 10.0 g CSO + 3.0 g S + different fillers.

**Figure 7:**
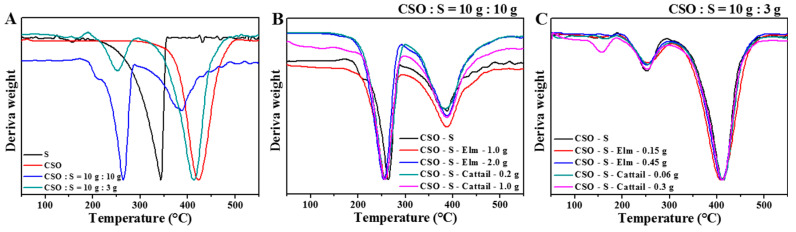
Derivative thermogravimetry (DTG) curves of sulfur, cottonseed oil (CSO) and bio-based polysulfide composites. (**A**) S, CSO, 10.0 g CSO + 10.0 g S, 10.0 g CSO + 3.0 g S, (**B**) 10.0 g CSO + 10.0 g S + elm/cattail, (**C**) 10.0 g CSO + 3.0 g S + elm/cattail.

Thermal stability of bio-based polysulfide composites were investigated by thermogravimetric analysis (TGA) and derivative thermogravimetry (DTG). Figure 6 and Figure 7A show that sulfur began to decompose at 200 °C, and completely decomposed at approximately 360 °C. The maximum weight loss temperature was 345 °C. While CSO began thermal decomposition at approximately 280 °C, and completely decomposed at approximately 510 °C. The maximum weight loss temperature was 425 °C. It can be seen that TGA and DTG curves of bio-based polysulfide composites prepared with different fillers were similar to those prepared without fillers. This may be due to the small contents of fillers did not affect the thermal decomposition of polysulfide composites. The polysulfide composites prepared in 10.0 g CSO + 10.0 g sulfur system showed a two-step thermal decomposition process, during approximately 200–290 °C and 290–520 °C, respectively (Figure 6A and Figure 7B). The maximum weight loss temperatures were 250 °C and 410 °C, respectively. The masses of polysulfide composites no longer decreased at around 520 °C, and the residual masses of polysulfide composites were about 15%. The polysulfide composites prepared in 10.0 g CSO + 3.0 g sulfur system also showed a two-step thermal decomposition process. The temperature range was consistent with the system of 10.0 g CSO + 10.0 g sulfur (Figure 6B and Figure 7C). But the maximum weight loss temperatures were different, 260 °C and 390 °C, respectively. The masses of polysulfide composites no longer decreased at around 520 °C as well, but the residual masses of polysulfide composites were only about 5%. 

Bio-based polysulfide composites exhibited a two-step thermal decomposition process. The first thermal degradation of bio-based polysulfide composites were due to decomposition of polysulfide composites. The degradation may be a depolymerization process through S-S bond cleavage [14]. This was supported by the endothermic peak in the DSC thermograms (Figure 5). The second mass loss was the thermal decomposition of the residual CSO in polysulfide composites with the increase of temperature [32]. The two-step thermal decomposition process was as a result of the unreacted sulfur and residual CSO present in polysulfide composites influenced the kinetics of their thermal decomposition. The mutual dispersion and distribution of raw material sulfur and CSO in polysulfide composites were unknown on micromorphological level. Therefore, the TGA curves in Figure 6 exhibit non-stable kinetics. Additionally, the chemical structures of polysulfide composites were not well defined. Molecular mass and its distribution, sulfur/CSO comonomer ratio and structure may vary considerably, additionally influenced the kinetics of decomposition [10]. TGA curves show that the residual mass of elemental sulfur and CSO were minimal and had been decomposed completely. In the same system, the residual masses of bio-based polysulfide composites were basically the same. This indicated that the thermal properties of polysulfide composites prepared in the same system were similar. However, the residual masses of polysulfide composites in 10.0 g CSO + 10.0 g sulfur system were about 15%, while the residual masses of polysulfide composites in 10.0 g CSO + 3.0 g sulfur system were about 5%. The higher sulfur contents of the polysulfide composites, the larger residual masses of the materials. This was due to the residue materials of polysulfide composites were consists of porous carbon doped with the remaining sulfur atoms [10]. Besides, the newly generated chemical bonds of polysulfide composites, such as C-S bonds, improved the thermal stability compared with the raw material sulfur and CSO. Moreover, bio-based polysulfide composites macromolecules presumably underwent fragmentation due to the reversibility of widely occurred S-S bonds at elevated temperatures, and formed shorter linkages that were more thermally stable. In that, after extremely high temperature exposition sulfur remained [51]. In general, the addition of fillers did not affect the thermal stability of bio-based polysulfide composites.

#### 3.2.4. FT-IR

**Figure 8:**
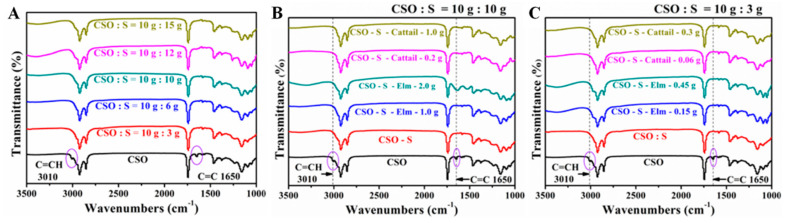
FT-IR spectra of CSO and bio-based polysulfide composites. (**A**) Different sulfur content, (**B**) 10.0 g CSO + 10.0 g S + different fillers, (**C**) 10.0 g CSO + 3.0 g S + different fillers.

The effects of sulfur and fillers content on chemical bonds of bio-based polysulfides were investigated in Figure 8. Figure 8A shows that FT-IR spectra of the five groups of polysulfides were consistent. Figure 8B,C presents that FT-IR spectra of the polysulfide composites prepared with different fillers in 10.0 g CSO + 10.0 g sulfur and 10.0 g CSO + 3.0 g sulfur system were consistent with those of polysulfide prepared without fillers. After polymerization, the signals of raw material CSO at ~3010 cm^−1^ of the C=C–H stretching vibrations and ~1650 cm^−1^ of the C=C stretching vibrations were disappeared. This indicated that the C=C double bonds in CSO reacted with sulfur. There were no new peaks at 2600–2550 cm^−1^, indicated that mercapto functional group (S-H) was probably not present in the structure of polysulfides and the composites. Wu and Valle et al. also found that mercapto functional group was not generated during inverse vulcanization of polysulfide [4,36]. The FT-IR spectra of the five groups of polysulfides (Figure 8A) and composites (Figure 8B,C) were identical, indicating that the chemical structures of them were similar. Both sulfur and fillers content did not affect the chemical structures of the polymers.

#### 3.2.5. Raman Spectra

**Figure 9:**
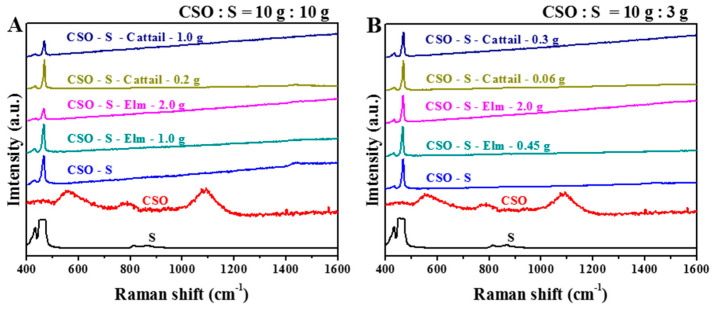
Raman spectra of sulfur, CSO, and bio-based polysulfide composites. (**A**) 10.0 g CSO + 10.0 g S + different fillers, (**B**) 10.0 g CSO + 3.0 g S + different fillers.

The chemical bonds of bio-based polysulfide composites were further investigated by Raman spectra. Figure 9 shows that Raman spectra of bio-based polysulfide composites prepared with different amount of fillers were basically the same in 10.0 g CSO + 10.0 g sulfur and 10.0 g CSO + 3.0 g sulfur system. The broad peaks at ~430 cm^−1^ and ~465 cm^−1^ were consistent with various symmetric and asymmetric stretching modes of S-S bonds. The results were compliance with those of Crockett, Worthington [14,32] and Mu [52] et al. The peaks of raw material CSO disappeared at ~560 cm^−1^, ~790 cm^−1^ and ~1090 cm^−1^, indicating that CSO was basically involved in inverse vulcanization reaction. Raman spectra also shows that no new chemical bonds were generated. The added fillers did not react with raw materials CSO, sulfur, and the products polysulfide composites.

### 3.3. Density Regulation of Bio-Based Polysulfide Composite

By adding fillers, elm and cattail, to CSO and sulfur system, density-adjustable bio-based polysulfide composites can be prepared via inverse vulcanization. The above results of polysulfide composites showed that the thermal properties and chemical structures of polysulfide composites with fillers were the same as those without fillers, indicating that no new chemical bonds were generated. But the surface morphologies of the polysulfide composites were different. Due to the different types and contents of fillers, the polysulfide composites with different densities were prepared by forming folds and holes of different sizes on the surface of the composites. These indicated that the added fillers were not chemically crosslink with CSO, sulfur, and polysulfide composites. The fillers were embedded in the polysulfide composites in the way of physical filling. Through heating and stirring, the fillers were dispersed in the reaction system during inverse vulcanization reaction. The fillers remained in the products when the polysulfide composites formed.

## 4. Conclusions

Density-adjustable bio-based polysulfide composites were designed and synthesized by inverse vulcanization of sulfur and CSO. The density regulation of polysulfide composite was achieved by using bio-based filler elm and cattail. Fillers were embedded in polysulfide composites in the way of physical filling. The polysulfide composites prepared in this study were tunable in density, so they have a broad application prospects in oilfield water plugging and profile control, marine oil spill accident treatment, and heavy metal removal in water bodies, etc. Our work provides an alternative and promising approach for preparing affordable density-adjustable bio-based polysulfide composites.

## Figures and Tables

**Table 1:** Effect of sulfur content on density and hardness of bio-based polysulfides.

Entry	CSO (g)	S (g)	Density (g cm^−3^)	Shore Hardness (Shore A)	Gelation Time (min)
1	10.0	2.0	-	-	-
2	10.0	2.5	1.01	-	120–130
3	10.0	3.0	1.02	4.5	60–65
4	10.0	4.0	1.08	12.3	50–55
5	10.0	6.0	1.12	19.5	40–43
6	10.0	8.0	1.12	28.9	38–42
7	10.0	10.0	1.27	29.3	30–33
8	10.0	12.0	1.27	29.4	28–30
9	10.0	15.0	1.34	30.0	24–26

**Table 2:** Effect of fillers on the density of bio-based polysulfide composites.

Entry	CSO (g)	S (g)	Filler		Density (g cm^−3^)
			Material	Content (g)	
1	10.0	10.0	No Filler	0	1.27
2	10.0	10.0	Active Carbon	1.0	1.26
3	10.0	10.0	Active Clay	1.0	1.29
4	10.0	10.0	300–800 CMC	1.0	1.25
5	10.0	10.0	800–1200 CMC	1.0	1.25
6	10.0	10.0	Elm	1.0	1.23
7	10.0	10.0	Cattail	1.0	1.16

**Table 3:** Elemental analysis of bio-based polysulfide composites.

Entry	CSO(g)	S(g)	Filler		Elemental			
			Material	Content (g)	S (%)	C (%)	H (%)	N (%)
1	10.0	10.0	No Filler	0	52.70	38.02	5.57	0.07
2	10.0	10.0	Elm	1.0	50.46	39.09	5.63	0.13
3	10.0	10.0	Elm	2.0	48.69	39.05	5.60	0.19
4	10.0	10.0	Cattail	0.2	52.35	39.15	5.57	0.10
5	10.0	10.0	Cattail	1.0	52.26	38.80	5.44	0.10
6	10.0	3.0	No Filler	0	23.31	59.49	8.48	0.05
7	10.0	3.0	Elm	0.15	22.98	59.86	8.50	0.12
8	10.0	3.0	Elm	0.45	22.57	59.90	8.50	0.07
9	10.0	3.0	Cattail	0.06	22.29	60.24	8.52	0.03
10	10.0	3.0	Cattail	0.3	21.98	59.82	8.45	0.07
